# Longitudinal stability of rapid and slow maxillary expansion

**DOI:** 10.1590/2176-9451.19.6.070-077.oar

**Published:** 2014

**Authors:** Fábio Henrique de Sá Leitão Pinheiro, Daniela Gamba Garib, Guilherme Janson, Roberto Bombonatti, Marcos Roberto de Freitas

**Affiliations:** 1 University Potiguar, Department of Dentistry, Associate professor, Department of Dentistry, University Potiguar; 2 University of São Paulo, School of Dentistry, Department of Orthodontics, Assistant professor. Department of Orthodontics, School of Dentistry - University of São Paulo/Bauru and Hospital for Rehabilitation of Craniofacial Anomalies/USP; 3 University of São Paulo/Bauru, School of Dentistry, Department of Orthodontics, Full professor, Department of Orthodontics, School of Dentistry - University of São Paulo/Bauru; 4 Paranaense University, Postgraduate program in Orthodontics, Professor, Postgraduate program in Orthodontics, Paranaense University (UNIPAR)

**Keywords:** Palatal expansion technique, Orthodontics, Treatment outcomes

## Abstract

**OBJECTIVE:**

The aim of this retrospective study was to compare the longitudinal stability of two types of posterior crossbite correction: rapid maxillary expansion (RME) and slow maxillary expansion (SME).

**METHODS:**

Study casts of 90 adolescent patients were assessed for interdental width changes at three different periods: pretreatment (T_1_), post-treatment (T_2_) and at least, five years post-retention (T_3_). Three groups of 30 patients were established according to the treatment received to correct posterior crossbite: Group A (RME), group B (SME) and group C (control- Edgewise therapy only). After crossbite correction, all patients received fixed edgewise orthodontic appliances. Paired t-tests and one-way ANOVA were used to identify significant intra and intergroup changes, respectively (P < 0.05).

**RESULTS:**

Except for intercanine distance, all widths increased in groups A and B from T_1_ to T_2_. In the long-term, the amount of relapse was not different for groups A and B, except for 3-3 widths which showed greater decrease in group A. However, the percentage of clinically relapsed cases of posterior crossbite was similar for rapid and slow maxillary expansion.

**CONCLUSION:**

Rapid and slow maxillary expansion showed similar stability in the long-term.

## Abstract (pt)

**OBJETIVO:**

o objetivo desse estudo retrospectivo foi comparar a estabilidade em longo prazo em dois tipos de correção da mordida cruzada posterior, sendo a expansão rápida (ERM) e a expansão lenta da maxila (ELM).

**MÉTODOS:**

modelos de estudos de 90 pacientes adolescentes foram avaliados quanto às alterações na largura interdentária em três diferentes tempos: pré-tratamento (T_1_), pós-tratamento (T_2_) e pelo menos cinco anos pós-contenção (T_3_). Três grupos de 30 pacientes foram definidos de acordo com o tratamento realizado para a correção da mordida cruzada posterior: Grupo A (ERM), grupo B (ELM) e grupo C (controle - apenas tratamento com técnica Edgewise). Após correção da mordida cruzada, todos pacientes receberam aparelhos ortodônticos fixos corretivos Edgewise. Teste *t* pareado e análise de variância a um critério (ANOVA) foram realizados para identificar alterações significantes intra- e intergrupos, respectivamente (p < 0,05).

**RESULTADOS:**

exceto para a distância intercaninos, todas as larguras aumentaram nos grupos A e B de T_1_ para T_2_. Em longo prazo, a quantidade de recidiva não foi diferente para os grupos A e B, exceto para a largura 3-3, que apresentou uma maior diminuição no grupo A. Clinicamente, entretanto, a porcentagem de casos com recidivas da mordida cruzada posterior foi semelhante para expansões rápida e lenta da maxila.

**CONCLUSÃO:**

Expansões rápida e lenta da maxila apresentaram estabilidades semelhantes em longo prazo.

## INTRODUCTION

Studies assessing longitudinal stability of rapid maxillary expansion have reported variable results.^1^^-^ 7 In general, maxillary base width changes remain quite stable, and only a slight amount of relapse is observed in the long-term.^1^^,^ 3 ^,^ 5 Krebs,^8^ in a classic study with implants, reported a decrease in maxillary bone width of just 0.5 mm right after the expansion retention period. This author also observed an increase after this period, but explained later that this was consequent to growth changes. After 20 years of follow-up, Haas^5^ reported no decrease in the width of the maxillary base and the nasal cavity in 10 of his studied cases. Cameron et al^3^ reported gain of 2 mm in maxillary bone width compared to a control group 6 years from the end of orthodontic treatment. On the other hand, dental arch transverse dimensions showed a more pronounced relapse according to some longitudinal studies, maintaining approximately 40% of initial molar expansion with significant difference from control.^2^^,^ 3 ^,^ 6

Studies on slow expansion also show variable results, although most of them have reported good longitudinal stability when performed in the mixed and permanent dentitions.^9^^-^ 15 While good stability of rapid maxillary expansion might be related to orthopedic effects, some authors associate the good stability of slow expansion with the maintenance of sutural integrity and stimulation of bone neoformation.^9^^-^ 15

Only a few studies actually compared slow and rapid maxillary expansions longitudinally.^16^^,^ 17 ^,^ 18 All of them used the expanded inner bow of the face bow appliance as the slow expansion device and the experimental groups did not present posterior crossbite. Expansion was performed to solve crowding in Class I patients^16^ and decompensate maxillary arch constriction in Class II patients previously to facial orthopedics.^17^^,^ 18 Herold^19^ compared the stability of expansion using Hyrax expander, quad-helix and removable plate (with coffin springs or expansion screw), in patients with posterior crossbite. Five years after treatment, relapse of posterior crossbite was greater in the quad-helix group compared to Hyrax and removable appliance groups. Given the variability in sample characteristics and the diversity in the results of these previous studies, differences in longitudinal stability between rapid and slow expansion in patients with posterior crossbite still need further clarification. This study aimed at comparing the longitudinal stability of rapid and slow maxillary expansion by means of assessing a sample at least five years after the end of orthodontic treatment. The following null hypothesis (H0) was formulated: There is no statistically significant difference between rapid and slow maxillary expansion stability in the long-term.

## MATERIAL AND METHODS

## Sample

In this retrospective cohort study, three groups of patients were analyzed. Sample size calculation was based on an internal pilot study. Based on type I error probability at 0.05 and a power of 0.80, 28 patients were required in each group.

Group A comprised 30 Caucasian patients (9 females; 21 males) with skeletal unilateral posterior crossbite^18^^,^ 20 and an initial mean age of 12.7 ± 1.2 submitted to rapid maxillary expansion (RME) and orthodontic treatment with fixed Edgewise appliance. Haas expander screw was activated one quarter of a turn in the morning and one quarter of a turn in the evening until 3 mm of overcorrection was obtained. Average expansion (transverse dimension with greater increase in each patient) was 4.91 ± 1.54. The appliance remained passive in place as a retainer for at least three months,^6^^,^ 21 ^,^ 22 after which a removable retention plate was installed and used for three months associated with Edgewise appliances.

Group B comprised 30 Caucasian patients (8 females; 22 males) with dental unilateral posterior crossbite^23^^,^ 24 and initial mean age of 13.7 ± 5.2 years submitted to slow maxillary expansion (SME) and orthodontic treatment with Edgewise appliances. Expanded levelling and alignment archwires associated or not with expanded inner-bow of Class II extraoral appliances were used for slow expansion. Average expansion was 3.78 ± 2.15. In groups A and B, archwire diagrams were defined after maxillary expansion.

Group C (control) comprised 30 Caucasian patients (17 females; 13 males) with an initial mean age of 13.0 ± 1.5 without posterior crossbite and treated with Edgewise mechanics alone. Table 1 describes the characteristics of each group.

**Table 1.** Descriptive statistics of the three groups.

	Group A	Group B	Group C	Intergroup comparison	
	(n = 30)	(n = 30)	(n = 30)		
Sex distribution	♀ 9 ♂ 21	♀ 8 ♂ 22	♀ 17 ♂ 13	X^2^	
				A-B-C A-B B-C A-C	p = 0.03* p = 0.77 p = 0.02* p = 0.04*
Initial mean age ± SD	12.7 ± -1.2	13.79 ± 5.23	13.03 ± 1.5	ANOVA p = 0.39	
Cases with maxillary premolar extraction (n)	4’s: 13 5’s: 0	4’s: 17 5’s: 1	4’s: 14 5’s: 2	X^2^ p = 0.43	
Molar relationship (%)	I - 10 II - 83.34 III - 6.66	I - 20 II - 70 III - 10	I - 13.33 II - 86.67 III - 0.00	X^2^ p = 0.40	

4´s, first-premolar; 5´s, second-premolar;

* statistically significant at P < 0.05.

The inclusion criteria for the experimental groups were: No previous orthodontic treatment; enough compliance with the removable appliances; adequate orthodontic finishing (correct anteroposterior, transverse and vertical inter-arch relationships, and absence of crowding or spacing);^25^ and at least six months of use of a maxillary Hawley retainer after the end of treatment. Both extraction and non-extraction cases were included. The entire treatment (expansion and fixed appliance treatment) lasted an average of 1.98 ± 0.73 years in group A, 2.23 ± 0.46 years in group B and 2.17 ± 0.74 years in group C.

Maxillary and mandibular study casts of 90 patients were obtained before treatment (T_1_), immediately after removing the Edgewise appliances (T_2_) and at least five years post-retention (T_3_). Mean post-retention period was 8.0 ± 2.77 in group A; 11.76 ± 5.00 years in group B; and 10.7 ± 4.96 years in group C. When patients returned to have the last impressions taken (T_3_), an examination in centric occlusion was carried out to clinically assess longitudinal stability. Posterior transverse correction was judged as unstable when edge-to-edge buccolingual relationship or posterior crossbite were observed.

In order to measure 3-3 (intercanine), 4-4 (interfirst-premolar), 5-5 (intersecond-premolar) and 6-6 (intermolar) transverse distances, a single blinded examiner used a 0.3 mm pencil (Pentel/P215, Japan) to set reference points on the most cervical gingival aspect of the palatal surfaces of teeth. A digital caliper (Dentaurum/Beerendonk) was used to measure the distance between points. For 4-4 width, statistical analysis was performed with non-extraction cases, only. All patients had widths recorded at T_1_, T_2_ and T_3_. T_2_-T_1_ interval represents the amount of change occurring during treatment. T_3_-T_2_ interval indicates long-term post-retention changes. Positive values mean expansion. Negative values represent constriction or relapse.

## Error of the method

The error of the method was assessed by repeating measurements in 20 randomly selected study casts within a two-week interval between the first and second measurements. Systematic errors were calculated using paired t-tests at 5% significance level. Random errors were evaluated with Dahlberg's formula:^26^ S^2^ = d^2^/2n.

## Statistical analysis

Intergroup comparisons regarding sex proportion, number of extraction and non-extraction cases and the types of malocclusion were performed with chi-square tests. Intergroup comparison regarding initial mean age was conducted with one-way ANOVA.

All variables were quantitative and continuous and displayed normal distribution as assessed by Shapiro-Wilk test at 5%. Therefore, one-way ANOVA followed by Tukey tests were used to compare the groups at the initial stage (T_1_), and the changes during treatment and long-term post-treatment periods (T_2_-T_1_ and T_3_-T_2_, respectively). Paired t-tests at 5% were used to assess treatment and post-treatment changes (T_2_-T_1_ and T_3_-T_2_).

Chi-square test with Yates continuity correction was performed to compare the percentage of stable and relapsed cases between groups A and B. Results were considered significant at P < 0.05.

## RESULTS

Sex proportion was significantly different in group C in relation to the other groups (Table 1). Initial mean age and proportion between extraction and non-extraction cases and types of malocclusion were similar in the three groups. There was no significant systematic error and random errors were within acceptable limits, ranging from 0.19 mm (4-4 distance) to 0.33 mm (6-6 distance) (Table 2).

**Table 2.** Systematic and random errors in each interdental width.

Interdental width	First measurement Mean ± SD (mm)	Second measurement Mean ± SD (mm)	p-value	Dahlberg's
3-3 (n = 20)	23.06 ± 1.54	23.05 ± 1.57	0.91	0.28
4-4 (n = 11)	25.69 ± 1.37	25.72 ± 1.26	0.68	0.19
5-5 (n = 20)	28.83 ± 2.47	28.73 ± 2.58	0.21	0.25
6-6 (n = 20)	32.53 ± 2.63	32.69 ± 2.60	0.13	0.33

3-3, Intercanine; 4-4, interfirst-premolar; 5-5, intersecond-premolar; 6-6, intermolar.

## Pretreatment intergroup comparison (T_1_)

Groups A and B had similar initial transverse widths, but group A had significantly smaller 4-4, 5-5 and 6-6 widths than group C (Table 3).

## Intergroup treatment changes comparison (T_2_-T_1_)

During treatment, there were similar transverse changes in groups A and B, except for 5-5 width which showed greater increase in group A. Group A had significantly greater increases than the control group in 4-4, 5-5 and 6-6 widths while group B only had it in 4-4 width (Table 3).

**Table 3.** Intergroup comparison at the pretreatment phase as well as during treatment and long-term post-treatment periods (one-way ANOVA and Tukey test).

Pre-treatment (T_1_)				
Interdental width	Group A	Group B	Group C	p-value
	Mean ± SD (mm)	Mean ± SD (mm)	Mean ± SD (mm)	
3-3	25.25 (3.15)	25.36 (3.39)	26.15 (2.68)	0.54
4-4	23.68 (4.13)^A^	24.30 (2.40)^A.B^	26.50 (2.98)^B^	0.00*
5-5	27.86 (3.74)^A^	28.53 (1.94)^A.B^	30.58 (3.25)^B^	0.04*
6-6	31.82 (4.39)^A^	32.42 (2.55)^A.B^	34.26 (3.06)^B^	0.02*
Treatment changes (T_2_-T_1_)				
3-3	-0.37 (2.32)	-0.004 (3.28)	-0.93 (2.33)	0.48
4-4	4.44 (1.34)^A^	3.79 (2.92)^A^	0.87 (2.01)^B^	0.00*
5-5	3.09 (2.34)^A^	1.23 (2.94)^B^	-0.31 (2.27)^B^	0.00*
6-6	2.72 (1.77)^A^	1.79 (2.89)^A.B^	0.28 (2.94)^B^	0.00*
Long-term post-treatment changes (T_3_-T_2_)				
3-3	-1.37 (0.80)^A^	-0.75 (1.04)^B^	-0.66 (0.86)^B^	0.00*
4-4	-1.36 (0.97)^A^	-0.74 (0.88)^A.B^	-0.48 (1.03)^B^	0.03*
5-5	-1.11 (1.33)	-0.58 (1.45)	-0.27 (1.17)	0.27
6-6	-1.04 (1.17)	-0.50 (1.52)	-0.53 (3.06)	0.25

* Statistically significant at P < 0.05.

Different letters in the same interdental widths represent statistically significant differences at P < 0.05. 3-3, Intercanine; 4-4, interfirst-premolar; 5-5, intersecond-premolar; 6-6, intermolar.

## Intergroup post-treatment changes comparison (T_3_-T_2_)

Group A had significantly greater 3-3 width decrease in comparison to group B, and greater decrease in 3-3 and 4-4 widths than group C (Table 3).

## Intragroup treatment changes (T_2_-T_1_)

Except for distance 3-3, all widths in groups A and B increased significantly with treatment. In group C, there were no statistically significant changes within the same period of treatment time of experimental groups (Table 4).

**Table 4.** Intragroup comparison of treatment and post-treatment changes (T2-T1; T3-T2) at each interdental width.

Interdental width	T_1_ Mean ± SD (mm)	T_2_ Mean ± SD (mm)	T_3_ Mean ± SD (mm)	T_2_-T_1_ p-value	T_3_-T_2_ p-value
Group A					
3-3	25.25 ± 3.15	25.39 ± 2.51	24.02 ± 2.67	0.43	0.00*
4-4	23.68 ± 4.13	28.12 ± 4.12	26.76 ± 4.21	0.00*	0.00*
5-5	27.86 ± 3.74	31.02 ± 3.99	29.90 ± 4.14	0.00*	0.00*
6-6	31.82 ± 4.39	34.54 ± 4.71	33.50 ± 4.81	0.00*	0.00*
Group B					
3-3	25.36 ± 3.39	25.23 ± 1.53	24.47 ± 1.62	0.99	0.00*
4-4	24.30 ± 2.40	28.20 ± 1.51	27.46 ± 1.61	0.00*	0.01*
5-5	28.53 ± 1.94	30.17 ± 3.41	29.58 ± 3.25	0.04*	0.04*
6-6	32.42 ± 2.55	34.45 ± 3.08	33.94 ± 2.89	0.00*	0.08
Group C					
3-3	26.15 ± 2.68	25.17 ± 1.46	24.51 ± 1.93	0.06	0.00*
4-4	26.50 ± 2.98	27.26 ± 1.90	26.78 ± 2.53	0.11	0.08
5-5	30.58 ± 3.25	30.15 ± 2.72	29.88 ± 3.11	0.49	0.24
6-6	34.26 ± 3.06	34.54 ± 2.88	34.01 ± 3.12	0.60	0.05

* Statistically significant at P < 0.05;

3-3, Intercanine; 4-4, interfirst-premolar; 5-5, intersecond-premolar; 6-6, intermolar.

## Intragroup post-treatment changes (T_3_-T_2_)

Group A evinced statistically significant decrease in all transverse distances, whereas in group B the 6-6 width was the only one which did not decrease significantly. In group C, the 3-3 width was the only one with statistically significant reduction (Table 4).

## Clinical assessment of crossbite correction stability

Clinically, crossbite correction was found to relapse in 20% of patients in group A, and in 30% in group B, but this difference was not significant (Table 5).

**Table 5.** Comparison between the number of stable and relapsed cases in experimental groups A and B according to clinical evaluation (chi-square test with Yates continuity correction).

	Groups		
	Group A	Group B	Total
Stable cases	24 (80%)	21 (70%)	45 (75%)
Relapsed cases	6 (20%)	9 (30%)	15 (25%)
Total	30	30	60

Yates Continuity Correction = 0.36; Degree of freedom = 1; P-value = 0.55.

## DISCUSSION

## Methods

This retrospective study compared three experimental groups which were initially comparable regarding initial mean age, number of extraction cases and types of malocclusion (Table 1). Sex distribution was also similar in the groups treated with maxillary expansion, but the control group showed different sex distribution when compared to the experimental groups which may constitute a limitation of this study, since changes in arch width vary between males and females.^27^ Nevertheless, this would not affect inter-experimental group comparison, which is the most important issue in this investigation. Inclusion of extraction cases could also be regarded as a limitation of the study. Mesiodistal tooth movement can obscure transverse changes of the dental arch;^28^ however, the number of extraction cases was very similar between groups (Table 1).

Instead of repeated ANOVA measures, dependent t-tests were used to assess intragroup inter-stage changes because only T_2_-T_1_ and T_3_-T_2_ comparisons were considered important for this study. This proves statistically correct. Additionally, previous studies have also used t-tests for pair comparison even in the presence of more than two groups or two therapy phases.^29^^,^ 30

## Intergroup comparisons

At pre-treatment phase, it is clear that maxillary arch transverse dimensions were smaller in the RME group compared to control, except for the canine region (Table 3). Initial intercanine widths were similar in all study groups. Therefore, posterior crossbite in groups A and B were determined by reduced premolar and molar widths.

During treatment (T_2_-T_1_), the RME group showed greater increase of posterior arch widths in comparison to control (Table 3). Transverse increase was similar in both groups treated with expansion, except for the 5-5 width which showed greater increase in the RME group compared to the SME group. Interestingly, intercanine width remained stable during treatment in all three groups (Tables 3 and 4). These findings are in accordance with previous publications^31^^,^ 32 of which possible explanation is that posterior crossbite more frequently involves only premolars and molars. In fact, this study found a very low prevalence (6.66%) of crossbite including maxillary canines. For this reason, initial expansion in the experimental groups might have been neutralized by subsequent comprehensive orthodontic treatment.^33^ In other words, after expansion, fixed appliances might have coordinated maxillary with mandibular intercanine distances.

In the long-term post-treatment period, transverse arch width decreased at the canine, premolar and molar regions of all three groups (Table 3). Such a decrease, however, was greater in the RME group compared to the SME group for distance 3-3, and for 3-3 and 4-4 widths when compared to control (Table 3). Surprisingly, post-treatment reduction in 5-5 and 6-6 widths were similar in the experimental and control groups (Table 3). Non-treated subjects in fact show arch width reduction after completion of permanent dentition and maturation of the face and dentition.^34^^,^ 35 Orthodontically treated patients also show arch width reduction after removing the retainer.^36^^,^ 37 ^,^ 38 Studies by Little^36^^,^ 37 ^,^ 38concluded that regardless of treatment changes, mandibular 3-3 width decreases slowly and continuously with time.

In the RME group, the decrease observed in anterior arch width was greater than that of the control group and can be assigned to expansion relapse. These findings corroborate McNamara et al^6^ who treated patients by means of RME followed by Edgewise therapy and found that intercanine distance was the only maxillary arch width showing greater decrease after treatment in comparison to an untreated control group. Linder-Aronson and Lindgren^2^ also observed greater relapse in intercanine compared to intermolar distance. Five years after the end of the retention period, only 23% and 45% of initial expansion remained at canine and molar regions, respectively. Ferris et al^4^ found a non-significant relapse in intercanine distance after seven years of RME retention, while premolar and molar widths showed a significant decrease. Differences in these findings might be explained by lip bumper therapy performed in the mandibular arch during RME treatment.

Eight years after the end of orthodontic treatment, transverse changes in the maxillary arch were similar in RME and SME groups, except for intercanine width (Table 3). Additionally, when considering the frequency of cases showing crossbite relapse at T_3_, no difference was identified between groups (Table 5). Clinically, posterior crossbite relapsed in 20% of patients in group A and in 30% of patients in group B. These results corroborate previous studies which found similarity between rapid and slow maxillary expansion stability,^16^^,^ 17 ^,^ 18 even though patients comprising these studies did not present posterior crossbite initially. A previous study including patients with posterior crossbite also observed no difference in intercanine and intermolar width changes between rapid and slow expansion five years post-treatment.^19^

Given the fact that long-term results yielded by RME and SME were similar, we can assume that a certain degree of skeletal maxillary constriction can be compensated with buccal tipping of posterior teeth when correcting posterior crossbite. Further prospective and randomized studies comparing patients with posterior crossbite treated by means of RME and SME are necessary for a better understanding of maxillary expansion stability in the long-term.

## CONCLUSIONS

The null hypothesis (H0) was accepted:

1. Long-term rapid and slow maxillary expansion stability are quite similar. Significantly greater intercanine width decrease was observed in rapid maxillary expansion, only.
2. The percentage of clinically relapsed cases of posterior crossbite was similar for both rapid and slow maxillary expansion.
